# Correction of Left Ventricular Outflow Tract Obstruction Caused by Anomalous Papillary Muscle and Subaortic Membrane

**DOI:** 10.21470/1678-9741-2017-0046

**Published:** 2018

**Authors:** Mario Augusto Cray da Costa, Ana Caroline Wippich

**Affiliations:** 1 Universidade Estadual de Ponta Grossa (UEPG), Ponta Grossa, PR, Brazil.

**Keywords:** Aortic Valve/surgery, Cardiomyopathies, Papillary Muscles, Heart Valve Prosthesis Implantation

## Abstract

This paper presents a case study of a 30-year-old male patient with dyspnea on exertion had echocardiographic diagnosis of aortic subvalvar stenosis. Discrete mitral regurgitation and aortic valve dysplasia with mild to moderate insufficiency and hypertrophic cardiomyopathy were also noted. During surgery, a rare condition was identified: presence of papillary muscle anomaly associated with the subaortic membrane as a cause of obstruction of the left ventricular outflow tract. With the resection of these structures and a mitral valve annuloplasty, the patient evolved with a significant improvement of clinical condition and heart failure, with no residual mitral insufficiency.

**Table:** 

Abbreviations, acronyms & symbols		
**CABG**		**= Coronary artery bypass grafting**
**ECG**		**= Electrocardiography**
**LAD**		**= Left anterior descending artery**
**LCA**		**= Lateral costal artery**
**LITA**		**= Lateral internal thoracic artery**
**SCA**		**= Subclavian artery**

## INTRODUCTION

Papillary muscle anomaly has been described as a rare cause of left ventricular outflow tract obstruction and hypertrophic cardiomyopathy^[1]^. The diagnosis can be made clinically with imaging tests, such as transthoracic echocardiography and nuclear magnetic resonance imaging. However, even among experienced professionals, it can be difficult to identify this anomaly by these exams and the definitive diagnosis is made only during surgery^[2]^. Thus, it is important to report this anatomical alteration so that it is considered a differential diagnosis for obstruction of the left ventricular outflow tract and its consequences.

## CASE REPORT

RRF, male, 30 years old, who complained of dyspnea on average efforts since childhood, with no other associated symptoms, which showed improvement at rest. He did not present antecedent of angina pain and syncope. Smoker of 20 cigarettes a day since 15 years of age, without comorbidities or previous surgeries. At physical examination, cardiac auscultation identified normal rhythmic sound, two-click, with a holosystolic (3+/6+) audible murmur in aortic and mitral foci, with irradiation to the left axilla.

There was no carotid murmur, jugular swelling, hepatomegaly or lower limb edema. Other systems did not present alterations. The patient was admitted to the Cardiac Surgery Service for evaluation.

A transthoracic echocardiogram was performed, which showed left ventricular hypertrophy with presence of subaortic membrane, determining medium/maximum left ventricular outflow tract gradient of 64/139 mmHg at rest.

Discrete mitral regurgitation and aortic valve dysplasia with mild to moderate insufficiency were also noted. The overall systolic performance of the left ventricle was preserved, with ejection fraction of 75% by the Teichholz method. In order to correct aortic subvalvar stenosis, the patient was referred to cardiac surgery via median sternotomy with cardiopulmonary bypass and intermittent cold blood cardioplegia, the cavas were cleared. Oblique aortotomy and transeptal access to the mitral valve were performed. During surgery, papillary muscle anomaly, with two supernumerary muscles inserted at the base of the anterior cusp of the mitral valve, obstructing the left ventricular outflow tract was identified. There was a subaortic membrane in the region of the interventricular septum and the aortic valve was slightly thickened and with prolapse of the non-coronary cusp. The papillary anomalous muscles and the subaortic membrane were resected through aortotomy after soft traction of the aortic valve cusps (Figures 1 to 4). Finally, a Carpentier 26 ring was implanted in the mitral valve by transeptal access. The patient left surgery hemodynamically stable, without vasoactive drugs, and was referred to the Intensive Care Unit of the Service, where he stayed for two days under intensive care and monitoring.

**Fig. 1:**
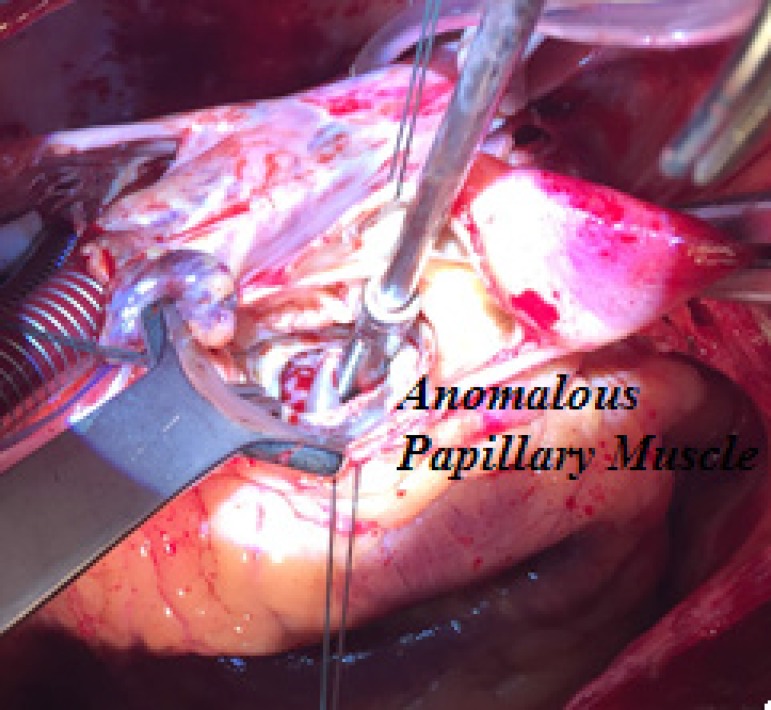
Surgical aspect.

**Fig. 4:**
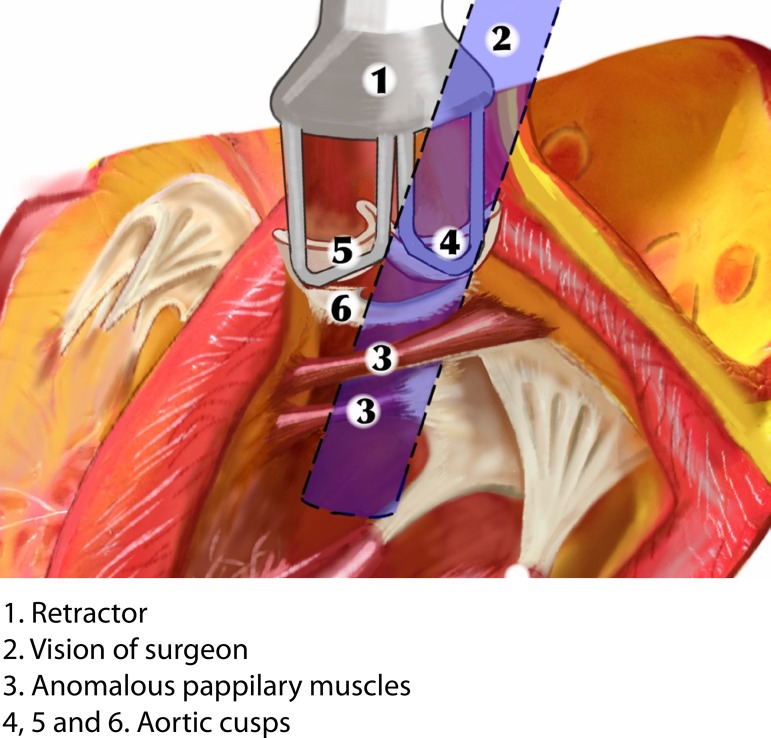
Schematic drawing. Vision of the surgeon.

After surgery the patient had an uneventful recovery and was discharged on the fifth postoperative day. At the time of hospital discharge, a new transthoracic echocardiogram was performed, which showed mild aortic insufficiency, absence of mitral regurgitation, and left ventricular outflow tract maximal gradient of 32 mmHg.

One month after surgery, a 24-hour Holter, which showed no significant changes, and a new transthoracic echocardiogram, which showed maintenance of mild aortic failure and absence of mitral regurgitation were performed. The left ventricular outflow tract maximal gradient was 16 mmHg. The patient remained asymptomatic. After six months, the patient remained symptom-free and a new echocardiogram revealed mild aortic and mitral valve thickening, maximum/median gradient through the aortic valve of 28/18 mmHg and through the mitral valve of 9/4 mmHg, without failure.

## DISCUSSION

Papillary muscle abnormalities are congenital anatomical changes identified in cases of hypertrophic cardiomyopathy. In order to explain a pathophysiology, Nomura et al.^[3]^ considered that an anomalous insertion of the papillary muscle is responsible for the obstruction of the left ventricular outflow tract and this may cause secondary concentric hypertrophy. Another mechanism of obstruction of the left ventricular outflow tract may be the anterior systolic movement of the mitral valve, caused by the asymmetric hypertrophy of the interventricular septum and also by the increased velocity of blood flow through a narrow path, known as the Venturi effect. Del Guzzo et al.^[4]^ raised the hypothesis that the constant turbulence of blood flow created by the obstruction coming from the anomalous papillary muscle could cause fibrosis and consequent aortic subvalvar stenosis.

Several papillary muscle abnormalities have been described. Minakata et al.^[2]^ described as intraoperative findings the presence of fusion of papillary muscle with the ventricular septum, fusion with a left ventricular free wall, the presence of accessory papillary muscle and, similar to that described on this report, insertion of papillary muscle directly at the anterior cusp of the mitral valve. More than one anomaly may be present in the same patient. The association of papillary muscle anomaly with subaortic stenosis is described as an even rarer event^[1,4]^.

Clinical findings in patients with papillary anomaly with left ventricular outflow tract obstruction include effort dyspnea on effort, angina, syncope, systolic murmur in aortic or accessory aortic focus^[1-4]^, as well as left ventricular hypertrophy and cardiomegaly identified in the 12-lead electrocardiogram and chest X-ray, respectively^[1,3]^. Nomura et al.^[3]^ reported an episode of ischemic stroke due to paroxysmal atrial fibrillation in a patient who had papillary muscle anomalies associated with hypertrophic cardiomyopathy and absence of stenosis in cerebral arteries.

Diagnosis can be made by imaging tests. The transthoracic echocardiogram, besides evidencing aortic and mitral regurgitation, allows the identification of mitral anomalies, as a continuity between the papillary muscle and the mitral valve. However, it should be emphasized that the recognition of papillary muscle abnormalities may be difficult even for experienced experts, being necessary incidences different from the common use experience, which consists of a longitudinal parasternal plane oriented towards the center of the left ventricle cavity^[2,5]^. Transesophageal echocardiography and a cardiac nuclear magnetic resonance are alternative imaging methods for diagnosis^[2]^. However, due to the difficulty of viewing the anomaly and the low index of suspicion, the diagnosis can be made during surgery.

Surgical correction consists of resection in the anomalous papillary muscle and, when present, aortic subvalvar stenosis^[1,6]^. Usually, these procedures are sufficient for clinical improvement for the patient. However, an associated myomectomy of the interventricular septum can be performed aiming at reducing the hemodynamic repercussions of hypertrophic cardiomyopathy^[1-3,5,6]^. The mitral valve should be preserved whenever possible, especially in young patients^[1,5]^. For this particular patient, the option was resection of accessory papillary muscles, resection of the subaortic membrane and mitral annuloplasty with Carpentier ring as a therapeutic strategy, in order to promote greater valve support. The evolution was optimal and the patient was asymptomatic, with competent valves and without significant gradient after 6 months of the surgery.

## CONCLUSION

According to literature review the association of papillary muscle anomalies with subaortic stenosis in hypertrophic cardiomyopathy is quite rare. Its identification by imaging test can be difficult, making the diagnosis only certain during surgery. Nuclear magnetic image and transesophageal echocardiography could be useful, but were not performed. Resection of the anomalous papillary muscle, associated with resection of aortic subvalvar stenosis, when present, is the treatment of choice. Placement of Carpentier's ring may be an alternative to optimize mitral valve function, since it should preferably be maintained.

**Table:** 

**Authors’ roles & responsibilities**	
MACC	Substantial contributions to the conception and design of the work; drafting the work or revising it critically for important intellectual content; final approval of the version to be published
ACW	Substantial contributions to the conception and design of the work; drafting the work or revising it critically for important intellectual content; final approval of the version to be published

## Figures and Tables

**Fig. 2:**
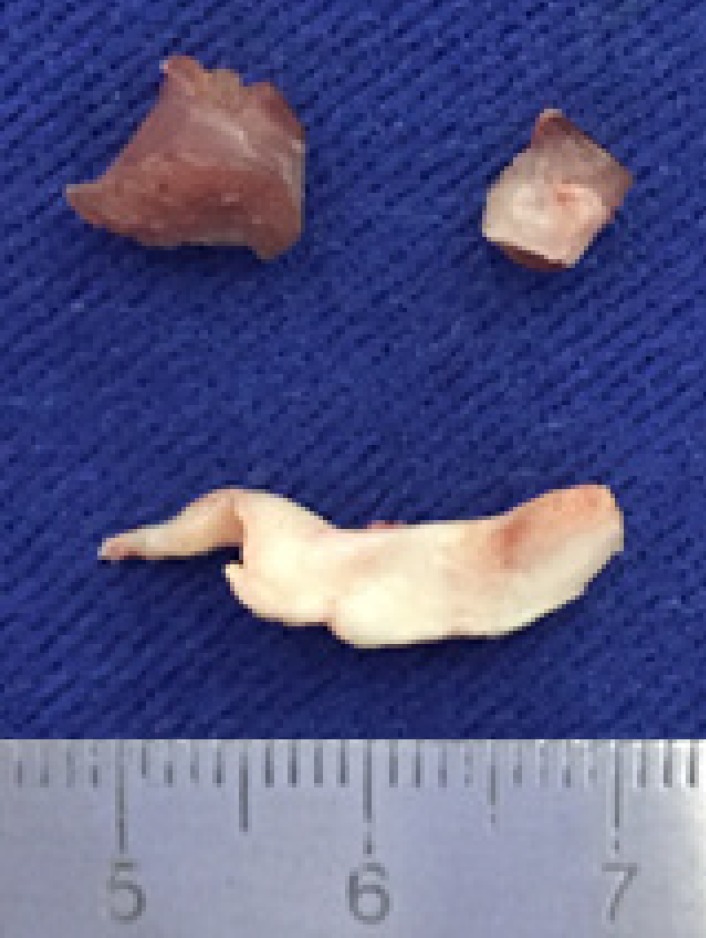
Subaortic membrane and papillary muscles.

**Fig. 3:**
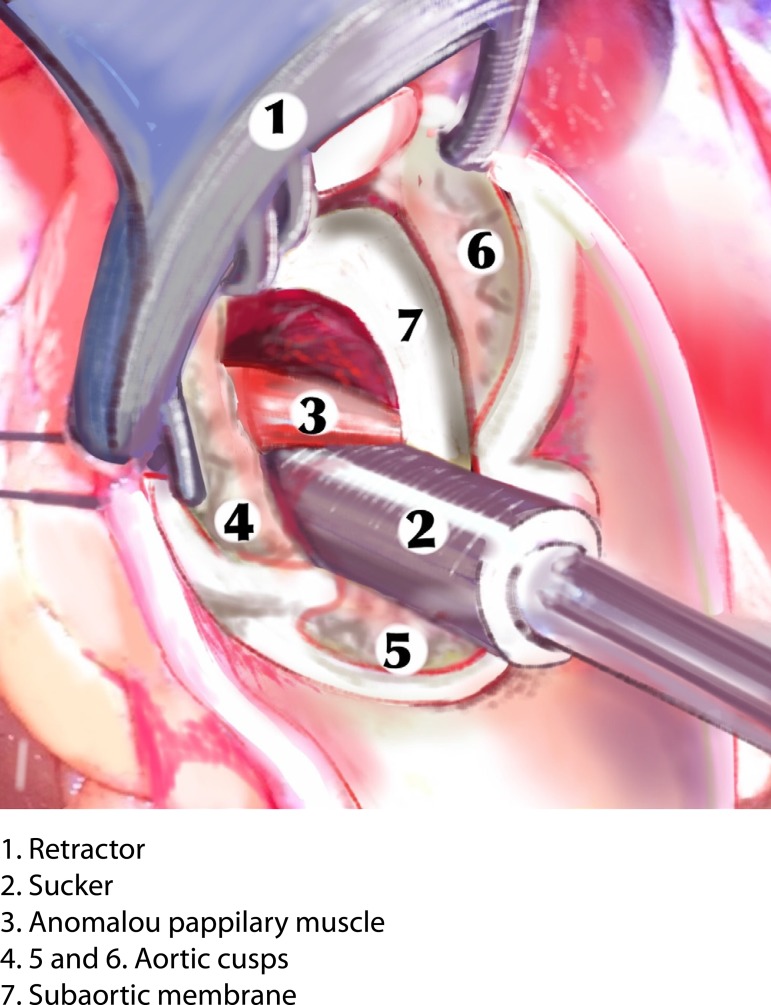
Schematic drawing. Vision of the surgeon.
